# Evaluating AI adoption challenges in healthcare using a Multi-Criteria Decision-Making approach: implications for predictive risk analytics

**DOI:** 10.3389/frai.2026.1827586

**Published:** 2026-05-20

**Authors:** Pulidindi Venugopal, Pratibha Garg, Chand Prakash, Sunil Kumar, Neha Gupta

**Affiliations:** 1 VIT Business School, Vellore Institute of Technology (VIT), Vellore, India; 2 Noida Business School, Amity University, Noida, India; 3 SGT University, Gurugram, India

**Keywords:** Analytic Hierarchy Process, artificial intelligence, data governance, decision-making trial and evaluation laboratory, healthcare analytics, Multi-Criteria Decision-Making, predictive risk analytics

## Abstract

**Introduction:**

Artificial intelligence (AI) adoption in predictive healthcare risk analytics can transform clinical decision-making and resource management; however, its implementation is limited by various socio-technical challenges.

**Methods:**

This study aims to identify and prioritize the key barriers influencing AI adoption using an integrated Decision-Making Trial and Evaluation Laboratory (DEMATEL) and Analytic Hierarchy Process (AHP) model. Based on a thorough literature review and expert validation, fifteen challenges were identified and categorized into five dimensions: technological, data-related, organizational, human/social, and ethical-regulatory. The DEMATEL method was used to analyse causal relationships among the challenges, while AHP was employed to determine their relative importance through hierarchical weighting.

**Results:**

The results indicate that the most influential structural drivers affecting adoption include data privacy and protection, data quality and completeness, lack of AI governance, and system interoperability, while leadership and strategic alignment emerge as critical organizational enablers. Data-related and governance-oriented challenges emerged as primary causal factors, whereas human-centred and ethical concerns predominantly appeared as dependent outcomes.

**Discussion:**

The study concludes that successful adoption of AI in predictive healthcare analytics requires strong leadership support, robust data governance systems, and transparent and interoperable technologies, and provides a structured roadmap for healthcare organizations to achieve scalable and reliable predictive analytics implementation.

## 1 Introduction

Artificial intelligence is transforming the landscape of modern healthcare by supporting data-driven and predictive clinical decision-making, promoting individualized medicine, and improving operational and administrative efficiencies within healthcare systems (Bajwa et al., 2021; Maleki Varnosfaderani and Forouzanfar, 2024). Predictive risk analytics has emerged as one of the most promising application areas. Using machine learning, natural language processing, and neural networks, AI-based predictive models can process multidimensional data from electronic health records (EHRs), medical imaging, wearable sensors, and genomic profiles to predict disease progression, identify high-risk patients, and make proactive actions (Dixon et al., 2024). The capabilities have significant potential to alleviate clinical burden, enhance patient outcomes, reduce hospitalizations, and streamline healthcare resources utilization.

Despite its transformative potential, the large-scale implementation of AI-powered predictive analytics in healthcare remains fragmented and uneven across global health systems. Even in healthcare setting where AI systems show superior predictive accuracy, conventional clinical environments still depend on clinician intuition, rule-based prognostication, or conventional statistical tools (Ahmed et al., 2023). This low adoption is not due to a lack of technological maturity but arises from a complex interplay of technical, organizational, ethical, legal and socio-behavioral challenges. Such multidimensional barriers not only hinder implementation, but also suppress the trust, acceptance, and the continued use of AI in routine clinical practice (Brandão, 2025).

The complexity of healthcare environments, heterogeneity of data structures, stringent regulatory requirements, and diversity of stakeholders further intensify these challenges. Challenges such as incomplete or biased data reduce the reliability and generalizability of predictive models, while technological barriers such as lack of interoperability, limited understanding of algorithms, and poor integration with legacy systems reduce clinician confidence. Organization constraints like insufficient infrastructure, lack of leadership support, high implementation cost, and the absence of training further obstruct adoption readiness. Additionally, ethical and regulatory issues such as fairness, accountability, patient consent, and data misuse concerns increase uncertainty and slow decision-making processes (Khan M. M. et al., 2025).

Existing research has mostly prioritized on improving the technical performance of AI models, stressing metrics such as accuracy, specificity and sensitivity. While these advancements have enhanced model effectiveness, relatively limited attention has been given to understanding the broader adoption ecosystem, and the interdependencies among various barriers influencing implementation. Furthermore, most available literature largely presents quite fragmented or descriptive analyses, lacking any systematic approach to evaluate causal relationships and prioritize challenges based on their overall impact on adoption readiness (Poon et al., 2025). This gap highlights the need for structured analytical frameworks capable of simultaneously examining interrelationships and relative importance of barriers. It is imperative to fill this gap for enabling evidence-based strategic planning, effective resource allocation, and informed policy formulation in AI-driven predictive healthcare analytics. Therefore, this study addresses the lack of integrated analytical frameworks that simultaneously examine causal interrelationships and prioritize AI adoption barriers in predictive healthcare analytics.

To fill this gap, this paper uses a hybrid Multi-Criteria Decision-Making (MCDM) framework integrating the DEMATEL and AHP to systematically identify, interrelate, and prioritize the critical challenges impacting AI adoption in predictive risk analytics within healthcare settings. DEMATEL enables the identification of cause–effect relationships among challenges, while AHP aids the prioritization of these challenges through hierarchical weighting. This integrated approach offers a thorough consideration of both the structural influence and relative importance of barriers, presenting deeper insights into the adoption ecosystem.

The study categorizes AI adoption challenges into five broad dimensions: (i) technological, (ii) data-related, (iii) organizational, (iv) ethical and regulatory, and (v) human and socio-behavioral. By incorporating these dimensions in the MCDM framework, the research progresses a holistic causal model that illustrates the complex interdependencies among these challenges and recognizes the most significant factors of AI adoption (Akbarighatar et al., 2023).

This study has threefold contributions. First, it contributes to theory through integration of socio-technical systems view with MCDM analytics, thus explaining the interdependent nature of AI adoption barriers in healthcare systems. Second, it makes the adoption of AI studies more methodologically rigorous by applying a hybrid DEMATEL–AHP model to decipher simultaneously both causality and prioritization of challenges. Third, it provides practical insights for healthcare practitioners, administrators, and policymakers by giving a structured prioritization of challenges to facilitate resource allocation, strategic decision-making, and policy development. On the whole, this study aims to enable the evolution from experimental AI applications to scalable, reliable, and socially responsible deployment in predictive healthcare analytics.

## 2 Literature review

The introduction of artificial intelligence (AI) into healthcare has witnessed significant increase over the past decade, and especially in the field of predictive risk analytics, in which machine learning models receive more and more application to predict disease progression, hospital readmissions, and population-level health trends (Faiyazuddin et al., 2025; Maleki Varnosfaderani and Forouzanfar, 2024). Although many studies emphasize the role of AI in complementing clinical decision-making and enhancing patient outcomes, the implementation of the innovations in laboratory environments to real world clinical practice remains inconsistent. The challenges faced by the complete implementation of AI-driven predictive systems in healthcare remain numerous and diverse, due to the technical constraints and insufficiency of data as well as the organizational resistance and ethical considerations (Khan M. M. et al., 2025; Amjad et al., 2023). This fragmented understanding of adoption barriers underscores the necessity for a structured analytical outlook to observe their interactions and relative influence.

In addition to the well-documented concerns related to data governance and technological readiness, another significant challenge listed by researchers is the uncertainty surrounding clinical liability, the absence of industry-wide standards to verify the validity of their algorithm, the absence of patient involvement in the AI system development, and the fear of overreliance on automated decision support (Morley et al., 2022; Jenko et al., 2025). Clinical practitioners often complain about lack of clarity in integration of artificial intelligence recommendations with medical judgment, mostly in high-risk situations where diagnostic accuracy is essential. Moreover, the healthcare systems in low- and middle-income countries face special contextual challenges such as infrastructural limitations, skills gaps, and resource constraints, which further make it more difficult to integrate AI at scale (Fahim et al., 2025).

Recognizing the complexity and interdependence of these challenges, scholars have made different attempts to classify and analyse them using models of readiness maturity or technology-organization-environment (TOE) or digital innovation theory (Khalil et al., 2026; N’Dri and Su, 2024). Nevertheless, these methods are not always able to give a clear precedence of barriers or analyze the cause-and-effect links between them. Based on these classifications, the present study classifies the identified barriers into five major dimensions: technological, data-related, organizational, ethical and regulatory, and human and socio-behavioral. These dimensions offer a structured foundation for examining the multidimensional barriers to the implementation of AI in predictive healthcare analytics.

### 2.1 Technological challenges

Technological issues denote the constraints that are associated with AI models and its incorporation with the current health information systems. An ongoing issue according to which there is no explainability and transparency of complex predictive models. Many innovative models, mainly deep learning models, work as “black boxes” which limit the clinicians’ ability to understand risk predictions and thereby lowering trust in AI-assisted decision-making (Holzinger et al., 2023; Rajpurkar et al., 2022). Explainability is now being considered as a requirement and not a feature in predictive risk analytics, where clinical accountability is the most important aspect.

Another key technology is interoperability. The distribution of healthcare data falls under heterogeneous systems, including electronic health records, laboratory systems, imaging repositories, and wearable devices. Poor levels of interoperability and outdated infrastructures hinder the smooth adoption of AI into real-time clinical workflows (Botha et al., 2024). Moreover, AI implementation at scale would need access to robust computational infrastructure, cloud systems capabilities, and solid cybersecurity systems which are often constrained especially in low- and middle-income countries (Hirani et al., 2024).

There is also a big problem with model generalizability. Trained predictive models can lead to worse performance when used with heterogeneous patient groups that are not representative of the areas where they were trained, which can decrease clinical reliability and equity (Rajpurkar et al., 2022). Such technological issues highlight the necessity for transparent, interoperable, and context-sensitive artificial intelligence systems. These constraints also directly influence data usability and organizational readiness, emphasizing their interconnected nature.

### 2.2 Data-related challenges

Data quality, availability, and governance, are essential aspects of the performance of AI-powered predictive analytics. According to the recent research, healthcare datasets remain incomplete, inconsistent, and biased systemically, which may be due to the fragmented documentation practices and the uneven data sources (Wei et al., 2025). These shortcomings have a direct impact on the accuracy and fairness of predictive risk models.

Data silos are still common in healthcare organizations, which restricts the building of longitudinal patient records that are critical in effective risk prediction. Fragmentation is also contributed by proprietary systems, inconsistent data standards, and limited data-sharing mechanisms (Babar et al., 2024). Moreover, the differences in clinical coding standards and terminologies complicate the data harmonization and cross-institutional model deployment.

Another significant barrier is privacy and security. The large-scale aggregation of sensitive patient data increases probability of unauthorized access and data breaches. Healthcare organizations are also burdened with more operation and legal responsibilities due to the compliance with the changing data protection regulations (World Health Organization, 2025). Therefore, the successful implementation of AI will necessitate advanced data governance systems that are capable of balancing between innovation and patient trust.

### 2.3 Organizational challenges

The success of AI adoption in healthcare is determined by the organizational readiness. Leadership commitment, strategic alignment, and adequate resource allocation are consistently presented as enablers of AI-driven transformation (Hradecky et al., 2022). Nevertheless, many healthcare institutions do not have formal AI governance policies, leading in fragmented pilot projects rather than holistic implementation strategies.

Financial constraints also pose significant barriers, as AI systems implementation requires significant investment into infrastructures, software, data management process, and specialized talent (Hassan et al., 2024). Such expenses are particularly prohibitive for small and medium-sized healthcare providers Also, the implementation of predictive risk analytics in the current clinical workflow can be associated with the need to redesign a process, train staff, and evaluate a system on a continuous basis, which can disrupt routine operations.

Resistance to change among clinicians and administrators and the absence of accountability between the human decision-maker and AI systems, further hinders adoption. Thus, organizational challenges extend beyond resource availability to include governance structures, institutional culture, and cross-functional coordination.

### 2.4 Ethical and regulatory challenges

The ethical and regulatory aspect is a unique critical aspect of AI implementation in healthcare. Algorithmic bias remains a significant concern, particularly when models are trained on unrepresentative or biased datasets, resulting in differences in the outcomes of disparate treatment among subgroups of patients (Hanna et al., 2025). Such biases have to be dealt with both technical interventions and thorough evaluation frameworks considering fairness and accountability.

Liability is another major concern. Even when predictions based on AI cause clinical errors, it is not clear whether the developer of the system, the service user, or the care facility should be held responsible (Cestonaro et al., 2023). This uncertainty make clinicians and administrators reluctant to adopt and rely on the AI systems.

In most jurisdictions, regulatory frameworks on AI in healthcare are at their infancy stages. Even though governments have started to send guidelines on AI approval, they encounter difficulties in regulating continuously learning models that develop after deployment (Palaniappan et al., 2024). Other ethical challenges include issues of informed consent, ownership of the data and autonomy of patients. These ethical and regulatory challenges significantly influence trust and acceptance among both clinicians and patients.

### 2.5 Human and socio-behavioral challenges

The role of human factors in the acceptance and implementation of the AI-based predictive systems is crucial. The perceptions of the models as accurate, transparent, and consistent with clinical intuition determine the trust of clinicians in AI (Yang et al., 2024). Cases in which AI recommendations overrules the professional judgment can increase the mistrust and minimize the use of the decision support system.

Healthcare providers can also be resistant to it due to fear of deskilling or fear of loss of professional autonomy (López-de-Arana Prado, 2025). To successfully integrate AI, it is necessary to frame it as the supportive tool that enhances clinical expertise instead of substituting it. Engagement of end-users during the system designing process and providing continuous training can further increase the user confidence and alignment.

At the patient level, data misuse concerns, perceived loss of personal connection in care delivery, and digital literacy may influence the trust and readiness to use AI-enabled services. It is important that such socio-behavioral determinants should be addressed using specific education and communication to ensure inclusive and equitable adoption.

### 2.6 Research gap

The reviewed literature shows that challenges to artificial intelligence adoption in predictive healthcare are multidimensional and highly interdependent, encompassing beyond purely technological constraints. While previous studies have acknowledged individual barriers, few research has systematically inspected their interrelationships or appraised their relative importance in influencing adoption readiness. This limitation highlights the necessity for analytical approaches capable of capturing both causal relationships and prioritization of challenges. To address this, the current research adopts a hybrid Multi-Criteria Decision-Making model integrating DEMATEL and AHP methods to provide a structured and comprehensive analysis of adoption barriers in predictive healthcare analytics.

## 3 Research methodology

### 3.1 Research design and rationale

In this study, a Multi-Criteria Decision-Making (MCDM) framework is used to conduct a systematic analysis and prioritization of the challenges that affect the adoption of AI-based predictive risk analytics in healthcare. Due to the multidimensional and interdependent nature of the technological, organizational, ethical, and behavioral barriers, a systematic analytical method capable of modelling the cause-and-effect relationship and relative significance is necessary. Traditional qualitative methods or single-criterion approaches are insufficient to address such complexity. MCDM techniques are particularly suitable for analysing complex decision-making contexts involving multiple interrelated criteria (Saaty, 2008; Govindan and Jepsen, 2016).

Among MCDM approaches, Decision-Making Trial and Evaluation Laboratory (DEMATEL) is used to identify the direction and strength of influence among factors and to model cause–effect relationships (Yazo-Cabuya et al., 2024). In parallel, the Analytic Hierarchy Process (AHP) facilitates hierarchical prioritization through structured pairwise comparisons (Chen W. et al., 2023). The integration of DEMATEL and AHP provides complementary analytical advantages: DEMATEL captures the structural interdependencies among challenges, while AHP determines their relative importance (Wu and Lee, 2007). This combined approach enables a comprehensive evaluation of both influence patterns and priority levels, thereby enhancing analytical rigor and managerial interpretability in identifying critical adoption barriers (Li et al., 2025). Although fuzzy extensions of DEMATEL and AHP have been proposed to address uncertainty in expert judgments, the present study adopts the classical versions of these methods to maintain methodological simplicity and interpretability. The selected expert panel possessed substantial domain expertise, ensuring consistency and reliability in evaluations, as reflected in acceptable consistency ratios. Furthermore, the use of a Delphi-based consensus approach helped to minimize subjectivity in responses (Shang, 2023). Future research may extend this framework by incorporating fuzzy or hybrid approaches to better capture uncertainty in complex decision-making environments.

### 3.2 Research framework

The research was conducted through a structured four-phase process:

#### 3.2.1 Phase I: identification of AI adoption challenges

A systematic review of peer-reviewed literature, policy documents, and industry reports was undertaken to identify key challenges associated with artificial intelligence adoption in predictive healthcare analytics. This was complemented by preliminary expert consultations to ensure practical relevance. Based on this process, fifteen critical challenges were identified and categorized into five dimensions: technological, data-related, organizational, ethical and regulatory, and human and socio-behavioral.

#### 3.2.2 Phase II: expert evaluation and data collection

A purposive sample of 15 experts was constituted, comprising healthcare practitioners (*n* = 5), artificial intelligence researchers and data scientists (*n* = 6), and policy and regulatory experts (*n* = 4). All participants possessed a minimum of 10 years of professional experience in digital health, healthcare analytics, or artificial intelligence implementation.

A two-round Delphi-based approach was employed to enhance the reliability of expert judgments. In the first round, experts validated and refined the identified challenges. In the second round, they provided pairwise comparisons and influence assessments for DEMATEL and AHP analyses using structured linguistic scales, which were subsequently converted into quantitative values.

The response rate for the expert survey was 100%, as all invited participants completed both rounds of evaluation. The structured design of the questionnaire ensured consistency across responses, while the iterative Delphi process facilitated consensus and improved the validity of the collected data.

#### 3.2.3 Phase III: DEMATEL analysis

The DEMATEL method was applied to analyse the causal relationships among the fifteen challenges and to classify them into driving (cause) and dependent (effect) groups based on their influence patterns.

#### 3.2.4 Phase IV: AHP prioritization

Subsequently, the Analytic Hierarchy Process was used to determine the relative weights of the identified dimensions and sub-criteria through pairwise comparisons. This enabled the hierarchical ranking of challenges based on their overall importance in influencing artificial intelligence adoption.

**Figure 1:**
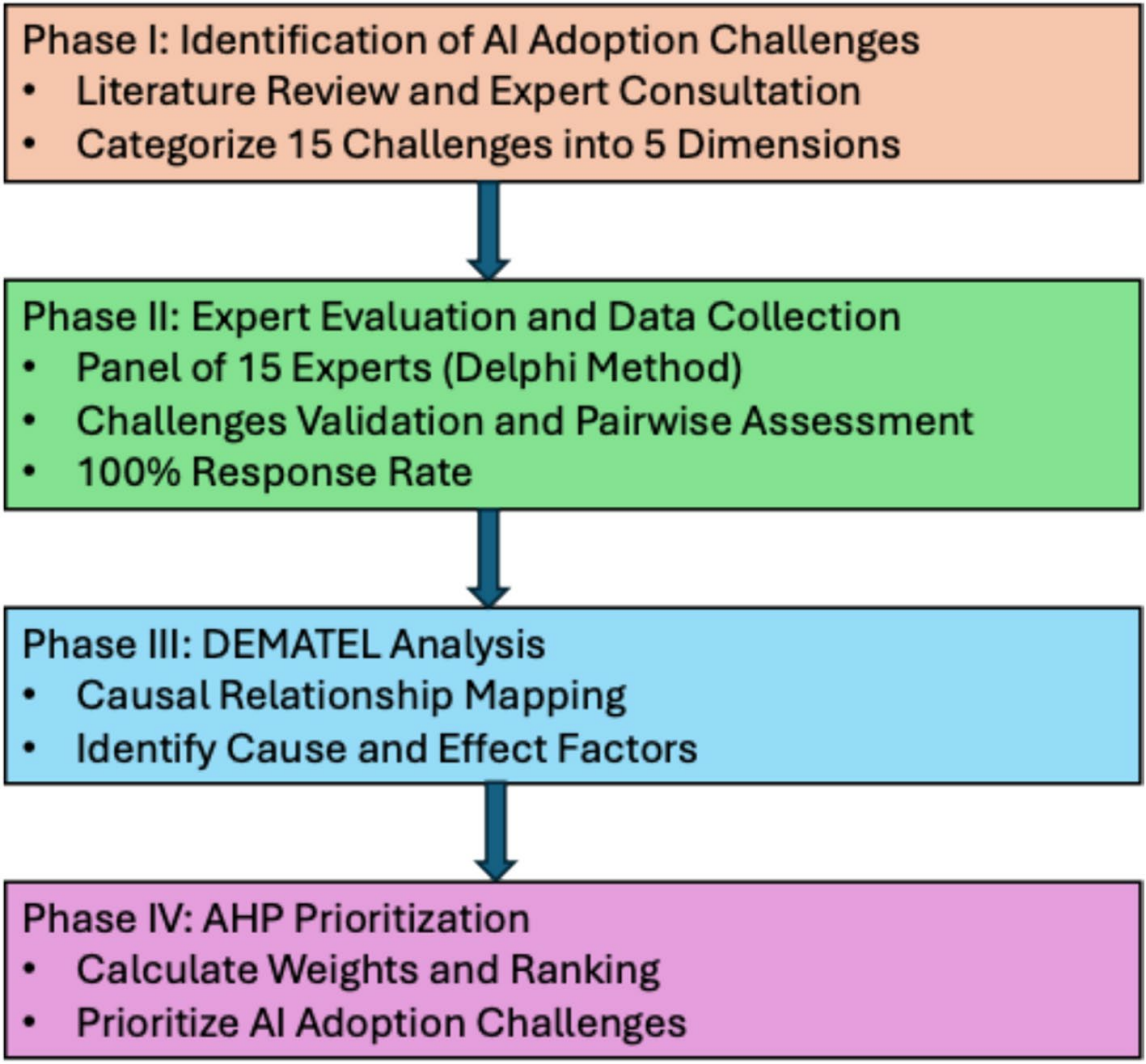
Research framework for DEMATEL–AHP analysis.

The overall research framework adopted in this study is illustrated in Figure 1.

### 3.3 Identification of challenges

The identification of AI adoption challenges in predictive healthcare risk analytics was conducted through a structured multi-stage process involving both a comprehensive literature review and expert validation. The objective was to develop a comprehensive yet manageable set of factors representing the technological, organizational, human, data-related, and ethical dimensions influencing AI implementation. First, peer-reviewed journal articles, conference proceedings, and recent industry reports were reviewed in systematic search to identify barriers to the introduction of AI integration in healthcare settings that are usually reported. Special focus was placed on the articles that covered the topic of predictive analytics, clinical decision support systems and digital health transformation efforts. The preliminary list of challenges based on the literature was examined by the domain experts to ensure contextual relevance, clarity of definition, and removal of redundancy. This iterative refinement process led to the identification of fifteen major challenges that fall under five overriding dimensions as shown in Table 1.

**Table 1:** Key challenges affecting AI adoption in predictive risk analytics.

Category	Challenge	Description	Source
Technological	T1. System interoperability	Lack of integration between legacy systems and AI platforms	Ahmed et al. (2023)
	T2. Algorithm transparency and explainability	Limited interpretability of AI decision models	Wang et al. (2025)
	T3. Scalability and performance limitations	Constraints in adapting models to diverse data volumes	Hradecky et al. (2022)
Data-related	D1. Data quality and completeness	Inconsistent or missing patient records affecting prediction	Khan M. M. et al. (2025)
	D2. Data privacy and protection	Lack of robust anonymization and consent frameworks	Khan M. M. et al. (2025)
	D3. Data standardization	Absence of unified data formats across hospitals	Ahmed et al. (2023)
Human and socio-behavioral	H1. Skill and knowledge gaps	Inadequate training for clinicians on AI systems	Alatawi and Kerari (2025)
	H2. Resistance to technology adoption	Behavioral reluctance due to perceived job displacement	Khan R. et al. (2025)
	H3. Ethical perception and patient trust	Concerns about privacy, accuracy, and accountability	Chen R. J. et al. (2023)
Organizational	O1. Infrastructure inadequacy	Limited computing and storage resources for AI deployment	Khan R. et al. (2025)
	O2. Financial constraints	High cost of AI hardware, software, and maintenance	Hradecky et al. (2022)
	O3. Leadership and strategic alignment	Weak executive support for digital transformation	Wang et al. (2025)
Ethical/regulatory	E1. Lack of AI governance	Unclear accountability frameworks for AI decisions	Elgin and Elgin (2024)
	E2. Algorithmic bias and fairness	Discriminatory outcomes from biased datasets	Chen R. J. et al. (2023)
	E3. Accountability and liability concerns	Ambiguity over responsibility for AI-driven errors	Khan M. M. et al. (2025)

### 3.4 DEMATEL method: analysis of causal relationships

The Decision-Making Trial and Evaluation Laboratory (DEMATEL) approach was used to explore the intricate interrelations among the identified AI adoption challenges (Tzeng and Huang, 2011). DEMATEL is specifically appropriate to examine cause-effect structures, in the socio-technical systems since it can represent both direct and indirect effects among the factors. Within the framework of predictive healthcare analytics, this strategy will allow identifying the key driving challenges that shape the ecosystem of the larger adoption. The computational process comprised of the following steps:

Step 1: Construction of the Direct-Relation Matrix

The panel of domain experts rated the level of influence among the fifteen challenges using a five-point scale ranging from 0 to 4 (0 = no influence, 4 = very high influence). Individual expert ratings were aggregated using arithmetic means to construct the initial direct-relation matrix.

Step 2: Normalization of the Matrix

The direct-relation matrix was normalized so that all the values were in the range [0, 1]. The transformation makes the numerical scale consistent and it makes it possible to make meaningful comparison across factors by avoiding an overbearing effect of one factor.

Step 3: Computation of the Total-Relation Matrix

The total-relation matrix was derived based on the normalized matrix and it includes direct and indirect effects among the challenges. This action will allow uncovering systemic relationships instead of disconnected pair relationships, as the adoption of AI in healthcare is interdependent.

Step 4: Derivation of Prominence and Relation Values

There were two important indicators calculated concerning each challenge:

- Prominence (D + R): Represents the overall degree of involvement of a challenge within the system, indicating its relative importance.
- Relation (D − R): Distinguishes causal (driving) factors from effect (dependent) factors.

   ○ The Positive values reflect causal challenges that cause influence on others.
   ○ Negative values refer to the challenges that are mostly affected by external factors.

#### 3.4.1 Mathematical formulation of DEMATEL

The DEMATEL method involves a series of matrix-based computations to derive causal relationships among factors. Let $X=[x_{\mathrm{ij}}]$ represent the initial direct-relation matrix obtained from expert evaluations, where $x_{\mathrm{ij}}$ indicates the degree of influence of factor *i* on factor *j*.

The normalized direct-relation matrix NNN is computed as:


$$N=\frac{X}{\max_{i}(\sum_{j}x_{\mathrm{ij}})}$$

The total-relation matrix *T* is then calculated as:


$$T=N{\left(1-N\right)}^{-1}$$

where *I* is the identity matrix.

The prominence and relation values are computed as:


$$D_{i}=\sum_{j}t_{\mathrm{ij}},\;\;R_{i}=\sum_{j}t_{\mathrm{ji}}$$

$$\text{Prominence}=D_{i}+R_{i}$$

$$\text{Relation}=D_{i}-R_{i}$$

A positive value of $\left(D_{i}-R_{i}\right)$ indicates a causal (driving) factor, while a negative value indicates an effect (dependent) factor.

### 3.5 AHP method: determining relative priorities

Following the identification of causal relationships with DEMATEL, the Analytic Hierarchy Process (AHP) was used to estimate the relative importance of each challenge. AHP is a structured Multi-Criteria Decision-Making technique that breaks down complex issues into hierarchical dimensions and employs pair-wise comparisons to be able to come up with quantitative weights (Saaty, 2008; Das and Sengar, 2022). The integration of AHP with DEMATEL permits to conduct the structural influence and hierarchical priority evaluation simultaneously. The process was divided into the following steps:

Step 1: Hierarchical Model Construction

A three-level hierarchy was developed:

- Level 1—Goal: Assessment and ranking of AI implementation issues in predictive risk analytics.
- Level 2—Criteria: Five dimensions, namely: Technological, Data-Related, Human/Social, Organizational, and Ethical/Regulatory.
- Level 3—Sub-Criteria: Fifteen potential challenges identified based on the literature review and validated by experts.

Step 2: Pairwise Comparison Matrices

Experts carried out pair-wise analysis based on the Saaty 1–9 scale where 1 indicates equal importance and 9 indicates extreme importance of one factor over another. Separate matrices were designed on criteria and sub-criteria levels.

Step 3: Weight Calculation and Consistency Verification

The comparison matrices were used to compute local priority weights using the eigenvector methods. Consistency Ratio (CR) was also determined to confirm rational consistency in expert judgments. Only matrices with CR ≤ 0.1 were accepted, ensuring reliability of the derived weights.

Step 4: Aggregation of Global Weights

Local weights of each challenge were then multiplied with the weight of its respective dimension to obtain the global weights. This consolidation generated a prioritized list of challenges, including their general importance in the context of AI adoption.

#### 3.5.1 Mathematical formulation of AHP

In the Analytic Hierarchy Process, pairwise comparison matrices are constructed to evaluate the relative importance of criteria. Let *A* = [*a*_*ij*] denote the comparison matrix, where *aija*_{*ij*}*aij* represents the relative importance of factor *i* over factor *j*.

The priority vector www is obtained by solving:


$$\mathrm{Aw}=\lambda_{\max}w$$

where $\lambda_{\max}$ is the maximum eigenvalue of matrix *A*.

The consistency of judgments is evaluated using the Consistency Index (CI) and Consistency Ratio (CR):


$$\mathrm{CI}=\frac{\left(\lambda_{\max}-n\right)}{n-1}$$

$$\mathrm{CR}=\frac{\mathrm{CI}}{\mathrm{RI}}$$

where *n* is the matrix size and RI is the Random Index. A value of CR ≤ 0.1 indicates acceptable consistency.

### 3.6 Integration of DEMATEL and AHP

The integration of DEMATEL and AHP provides a comprehensive analytical framework by combining causal analysis with hierarchical prioritization. DEMATEL is employed to identify the structure of interdependencies among challenges and to classify them into cause and effect groups based on their influence patterns. In contrast, AHP is used to determine the relative importance of each challenge through pairwise comparisons and weight computation.

The integration is implemented sequentially. First, DEMATEL establishes the causal relationships and identifies key driving factors within the system. Subsequently, AHP assigns quantitative weights to each dimension and sub-criterion, enabling prioritization of challenges based on their relative significance. The combined insights allow for identifying not only the most influential factors but also those that require immediate strategic attention. This hybrid approach enhances both analytical rigor and managerial relevance in evaluating AI adoption challenges.

## 4 Results and discussion

The integrated DEMATEL–AHP framework provides complementary insights about the interdependency of the structural and the hierarchical priorities of the problems of the AI adoption in predictive healthcare risk analytics. Whereas DEMATEL finds the cause-and-effect relationships and the patterns of systemic influence, AHP is able to measure the relative significance of each obstacle, allowing prioritization based on evidence.

### 4.1 DEMATEL results: causal relationship analysis

**Figure 2:**
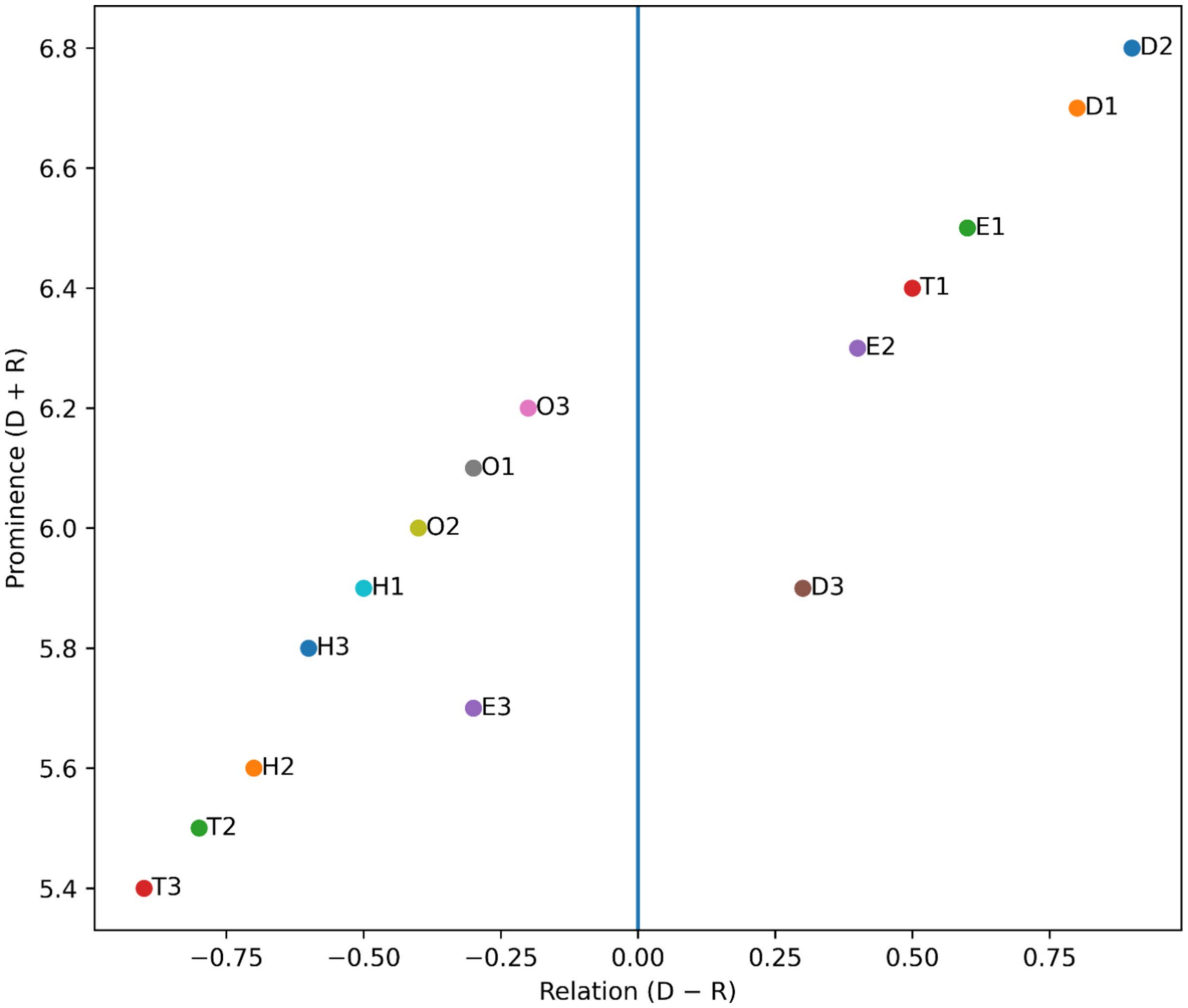
DEMATEL cause–effect diagram illustrating prominence (D + R) and relation (D − R) values of AI adoption challenges.

The DEMATEL analysis generated the total-relation matrix (Table 2) and the corresponding cause–effect diagram (Figure 2), illustrating the systemic interactions among the fifteen identified challenges. The prominence value (D + R) reflects the overall level of interaction of each factor within the system, whereas the relation value (D − R) distinguishes between causal (driving) and effect (dependent) challenges (Tzeng and Huang, 2011).

**Table 2:** DEMATEL prominence and relation values for AI adoption challenges.

Code	Challenge	D + R (Prominence)	D − R (Relation)	Group
D2	Data privacy and protection	6.82	+0.94	Cause
D1	Data quality and completeness	6.71	+0.87	Cause
E1	Lack of AI governance	6.52	+0.65	Cause
T1	System interoperability	6.48	+0.52	Cause
E2	Algorithmic bias and fairness	6.36	+0.41	Cause
O3	Leadership and strategic alignment	6.28	−0.12	Effect
O1	Infrastructure inadequacy	6.17	−0.25	Effect
O2	Financial constraints	6.11	−0.31	Effect
H1	Skill and knowledge gaps	6.05	−0.36	Effect
H3	Ethical perception and patient trust	5.98	−0.48	Effect
D3	Data standardization	5.86	+0.22	Cause
E3	Accountability and liability concerns	5.74	−0.17	Effect
T2	Algorithm transparency and explainability	5.62	−0.28	Effect
T3	Scalability and performance limitations	5.56	−0.34	Effect
H2	Resistance to technology adoption	5.42	−0.41	Effect

The cause–effect diagram (Figure 1) visually mapped these relationships, showing a clear directional influence from a directional influence from data, technological, and governance-related causes toward organizational, human, and ethical perception outcomes. This highlights that fostering an enabling infrastructure and governance culture precedes behavioral acceptance and regulatory alignment.

The findings indicate that data-related, technological, and ethical–regulatory factors predominantly occupy the causal group indicating their dominant influence on the adoption ecosystem. In particular, D2—Data privacy and protection, D1—Data quality and completeness, E1—Lack of AI governance, and T1—System interoperability demonstrated high positive relation values, highlighting their strong driving influence across the adoption ecosystem. These results align with prior research emphasizing that robust data governance and interoperable digital infrastructures are foundational to successful AI deployment in healthcare environments (Dwivedi et al., 2021; Wang et al., 2025).

Conversely, organizational and human–socio-behavioral factors emerged primarily as effect-type challenges. Factors such as O1—Infrastructure inadequacy, O2—Financial constraints, H1—Skill and knowledge gaps, and H3—Ethical perception and patient trust exhibited negative relation values, appear to be influenced by upstream governance and technological conditions. Previous studies have similarly shown that clinician trust and ethical acceptance often evolve as downstream outcomes once transparency, accountability, and data reliability improve (Chen R. J. et al., 2023; Alatawi and Kerari, 2025).

Overall, the DEMATEL findings emphasize that AI adoption challenges are not isolated; instead, they form a tightly interconnected socio-technical system. Addressing high-driving factors such as governance structures and data protection mechanisms is therefore likely to generate cascading improvements across organizational and human dimensions (Khan R. et al., 2025).

### 4.2 AHP results: hierarchical prioritization of challenges

Following the causal analysis, the AHP method was employed to quantify the relative importance of each challenge (Table 3). All pairwise comparison matrices achieved consistency ratios below the accepted threshold (CR ≤ 0.1), confirming the reliability of expert judgments.

**Table 3:** AHP-based prioritization of AI adoption challenges.

Category	Code	Challenge	AHP weight	Rank
Data-related	D2	Data privacy and protection	0.142	1
Data-related	D1	Data quality and completeness	0.136	2
Ethical–regulatory	E2	Algorithmic bias and fairness	0.122	3
Technological	T2	Algorithm transparency and explainability	0.106	4
Organizational	O3	Leadership and strategic alignment	0.095	5
Human	H3	Ethical perception and patient trust	0.089	6
Ethical–regulatory	E1	Lack of AI governance	0.085	7
Organizational	O1	Infrastructure inadequacy	0.079	8
Organizational	O2	Financial constraints	0.067	9
Human	H1	Skill and knowledge gaps	0.061	10
Data-related	D3	Data standardization	0.058	11
Technological	T3	Scalability and performance limitations	0.053	12
Technological	T1	System interoperability	0.048	13
Ethical–regulatory	E3	Accountability and liability concerns	0.041	14
Human	H2	Resistance to technology adoption	0.038	15

At the dimension level, data-related and ethical–regulatory challenges received the highest aggregated weights, underscoring the central role of trustworthy data ecosystems and governance mechanisms in AI adoption (Dwivedi et al., 2021). Among individual sub-factors, Data privacy and protection and Data quality and completeness ranked highest, reflecting increasing institutional emphasis on secure and reliable data infrastructures (Chen R. J. et al., 2023).

Algorithm transparency and explainability also emerged as a key priority, with Algorithm transparency achieving a strong global weight. This suggests that explainable AI models are critical for bridging technical capabilities with regulatory acceptance and clinician confidence (Wang et al., 2025). Leadership and strategic alignment were another highly ranked factor, reinforcing the argument that digital transformation initiatives require strategic vision and executive commitment rather than purely technical solutions (Khan M. M. et al., 2025).

Human-centered challenges, including Ethical perception and patient trust and Skill and knowledge gaps, received comparatively lower but still significant weights. Their positioning indicates that while behavioral acceptance is essential, it tends to improve once foundational governance and technological barriers are resolved (Alatawi and Kerari, 2025).

### 4.3 Integrated DEMATEL–AHP insights

The integration of DEMATEL and AHP provides a comprehensive perspective on AI adoption challenges. DEMATEL identifies structural causality, whereas AHP focuses on the perceived strategic significance of each challenge (Tzeng and Huang, 2011; Saaty, 2008). This distinction explains why certain factors, such as (O3—Leadership and strategic alignment), demonstrate high prominence in AHP despite appearing as effect-type variables in the DEMATEL structure.

#### 4.3.1 Leadership and data governance as foundational enablers

The findings highlight the presence of data-related and governance challenges, specifically, D2—Data privacy and data protection, D1—Data quality and completeness, E1—Lack of AI governance, as the key institutional priorities. All these aspects prove to be influential drivers (as shown by DEMATEL) as well as those with high relative weights (as calculated by AHP), which makes them very important leverage points in improving a system. Making data governance mechanisms stronger can result in both regulatory compliance, algorithm reliability, and institutional trust, creating ripple effects on an organizational and human dimensions, respectively, (Dwivedi et al., 2021; Chen R. J. et al., 2023).

#### 4.3.2 Interoperability and transparency as systemic bridges

Technological challenges such as (T1—System interoperability) and (T2—Algorithm transparency and explainability) bridge data infrastructure and acceptability among stakeholders. Explainable AI models reduce ambiguity, alleviate perceptions of bias and enhance compliance with regulations, which in turn foster clinician confidence and patient trust. These results confirm the opinion that transparency is not merely a technical attribute, but rather a strategic enabler of ethical and institutional legitimacy (Wang et al., 2025; Chen R. J. et al., 2023).

#### 4.3.3 Human and ethical concerns as downstream outcomes

Conversely, ethical, and human-centred challenges, such as resistance to adoption (H2), skill and knowledge gap (H1), Accountability and liability concerns (E3) appear comparatively downstream in both structural influence and the priority ranking. This indicates that, the acceptance of behavioral aspect is developed gradually as the governance, interoperability, and quality of data become better, a tendency that is also typical of social-technical transformation theories in healthcare digitalization (Khan M. M. et al., 2025; Alatawi and Kerari, 2025).

### 4.4 Sensitivity analysis

To evaluate the robustness and reliability of the results obtained from both the DEMATEL and AHP methods, a comprehensive sensitivity analysis (SA) was conducted. The objective was to examine how variations in input judgments and criteria weights influence the stability of causal relationships and priority rankings.

#### 4.4.1 Sensitivity analysis for AHP

**Figure 3:**
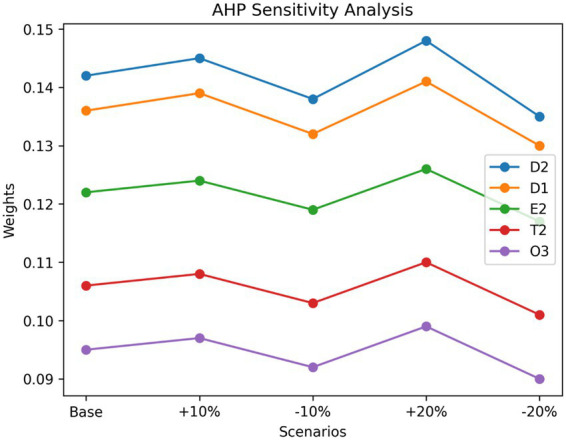
Sensitivity analysis of AHP weights using line diagram.

For AHP, sensitivity analysis was performed by introducing controlled variations (±10 and ±20%) in the weights of the main criteria (technological, data-related, organizational, ethical–regulatory, and human dimensions). The resulting changes in the ranking of sub-criteria were analyzed and visualized using line diagrams and radar charts (Figure 3) to enhance interpretability. The graphical analysis indicates that the top-ranked challenges-data privacy and protection (D2) and data quality and completeness (D1) remain consistently dominant across all scenarios. The radar chart further demonstrates minimal deviation in their relative importance, confirming their robustness as critical decision factors. Lower-ranked factors exhibited minor fluctuations under different weighting conditions; however, these variations did not significantly alter the overall prioritization structure. The line diagram clearly illustrates that the ranking trends remain largely invariant, particularly for high-impact factors, indicating strong consistency in expert judgments.

#### 4.4.2 Sensitivity analysis for DEMATEL

**Figure 4:**
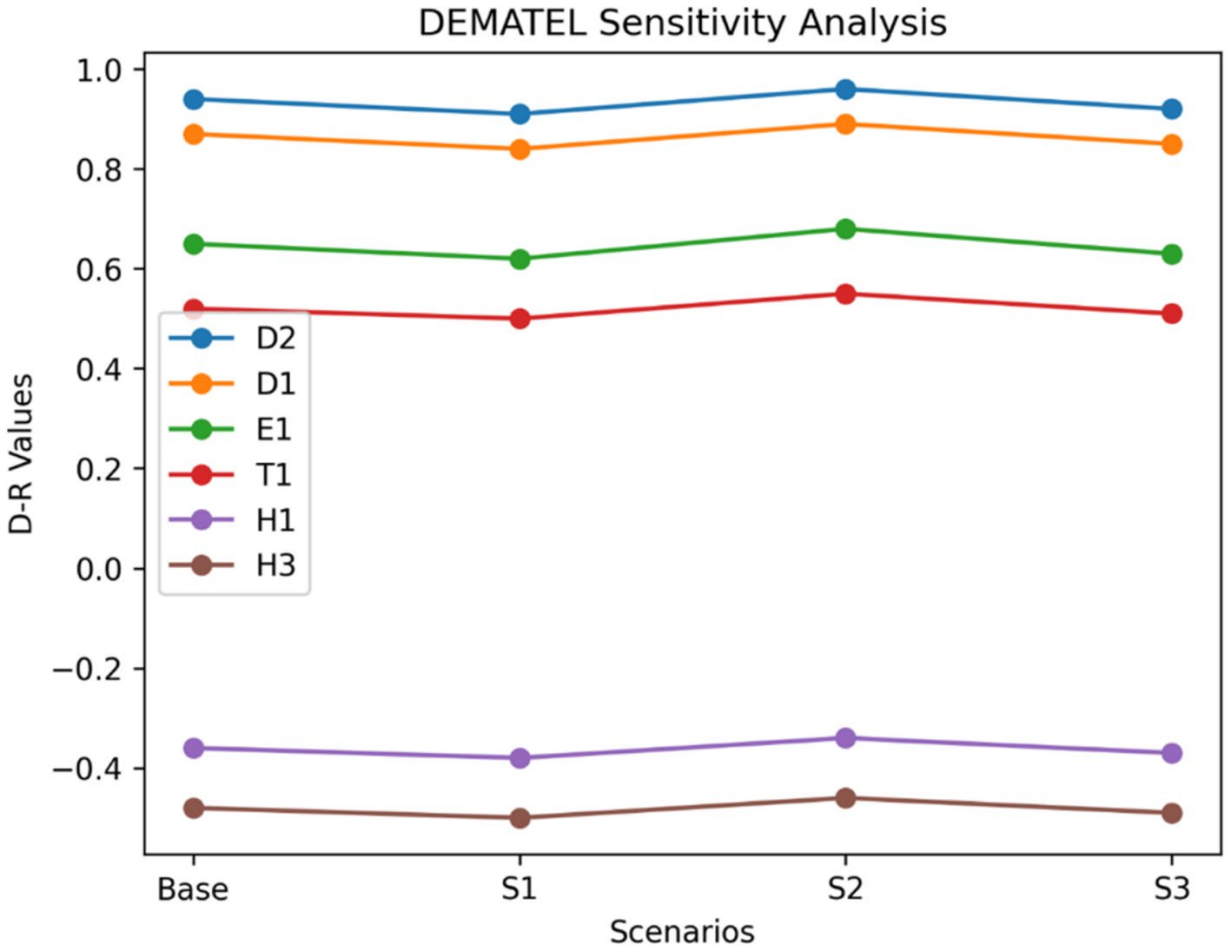
Sensitivity analysis of DEMATEL causal relationships.

For DEMATEL, sensitivity analysis was conducted by varying the threshold value used to construct the cause–effect diagram and by introducing slight perturbations in the normalized direct-relation matrix. The purpose was to assess the stability of causal group classification (cause vs. effect) and prominence values. The results, illustrated graphically in Figure 4, show that key driving factors such as data privacy and protection (D2), data quality and completeness (D1), and lack of AI governance (E1) consistently remain within the causal group across different scenarios. Similarly, dependent factors such as skill gaps (H1) and patient trust (H3) retain their classification as effect variables. Although minor variations were observed in the magnitude of prominence (D + R) and relation (D − R) values, the overall causal structure of the system remained stable. This confirms that the interdependencies identified through DEMATEL are robust and not highly sensitive to small changes in expert evaluations.

#### 4.4.3 Overall interpretation

The combined sensitivity analysis results from both DEMATEL and AHP confirm that the study findings are robust, reliable, and stable under varying conditions. The graphical representations (line and radar charts) enhance the interpretability of results by clearly demonstrating the consistency of key drivers and priority factors. These findings strengthen the validity of the integrated DEMATEL–AHP framework and reinforce confidence in the identified strategic priorities for AI adoption in predictive healthcare risk analytics.

## 5 Conclusion

This paper has used an integrated DEMATEL-AHP model to conduct a systematic assessment of those obstacles facing the uptake of AI in predictive healthcare risk analytics. The synthesized analysis showed that the implementation of AI is essentially a socio-technical change process influenced by interdependent technological and organizational factors (Dwivedi et al., 2021; Lee et al., 2023). Results of DEMATEL showed that governance structure, data privacy system, and interoperability are the main structural forces in the adoption ecosystem, and leadership alignment is a high-prominence organizational facilitator to downstream implementation. These findings are consistent with prior research emphasizing the role of governance and data readiness as foundational prerequisites for scalable AI adoption in healthcare (Chen R. J. et al., 2023; Wang et al., 2025).

The AHP prioritization also highlighted the overall importance of data privacy and protection, data quality and completeness, algorithm transparency, and leadership support as key factors to define the organizational readiness for AI integration. Existing literature suggests that explainable and trustworthy AI systems enhance clinician confidence and support regulatory compliance, thereby facilitating long-term adoption (Chen R. J. et al., 2023). Human-centered challenges, including Skill gaps and patient trust were identified to be heavily rely on the upstream governance and technological advancements of supporting the socio-technical character of the digital transformation in healthcare settings (Alatawi and Kerari, 2025; Khan R. et al., 2025).

From a theoretical perspective, this study is an addition to the existing amount of literature in that it provides an amalgamation of causal analysis and hierarchical prioritization as a comprehensive method of assessing the complex adoption barriers. Practically, the findings offer healthcare decision-makers a logical roadmap of prioritizing investments and policy ideas that would result in responsible AI implementation (Dwivedi et al., 2021).

Overall, the paper emphasizes that the implementation of AI sustainability within healthcare is not only technologically advanced but also requires governance maturity, the presence of data ecosystem, and ethical disclosure aspects equally emphasized in the modern research on digital health transformations (Wang et al., 2025).

## 6 Practical and policy implications

The results of the present work have some practical implications to the healthcare administrators, policymakers, and providers of AI solutions interested in speeding up the adoption of responsible AI in predictive risk analytics.

### 6.1 Enhancing digital governance and leadership

Formal AI governance structures must be institutionalized in healthcare organizations with roles, responsibilities and accountabilities being explicitly defined in clinical, technical and administrative functions. To match AI initiatives with the organizational strategy, strong leadership commitment is needed with the help of a coherent digital vision and commitment to it. Instead of being an upstream causal factor, leadership is an operational catalyst that allows the organizations to take the opportunities of data governance and technological readiness. Leadership-driven digital cultures further allow interdisciplinary collaboration among clinicians, data scientists, and IT specialists to reduce the fragmentation of the implementation.

### 6.2 Developing resilient data infrastructure and interoperability

Policymakers and health system leaders must prioritize investments in data standardization and interoperability. Interoperable electronic health record systems, standardized clinical terminologies, and secure data exchange mechanisms are foundational to reliable predictive analytics. At the system level, such advanced methods as federated learning may be backed by collaborative data-sharing ecosystems that allow model generalizability and at the same time protect patient privacy.

### 6.3 Cultivating algorithmic transparency and ethical assurance

The operationalization of explainable AI principles by AI developers and regulators should also be done in collaboration to improve clinician knowledge and trust in predictive models. Transparency, bias detection, and ethical audit mechanisms should be embedded throughout the AI development and deployment lifecycle. Regulatory authorities can also contribute to adoption by providing certification and assessment systems of reliable AI systems, which focus on safety, fairness, and responsibility. Improving Multidisciplinary Skills and Workforce Capacity.

### 6.4 Improving multidisciplinary skills and workforce capacity

AI literacy should be integrated into medical education, clinical training, and continuous professional development programs. Healthcare professionals need basic skills in data analysis, model verification, and responsible use of AI to interact confidently with predictive systems. Multidisciplinary training initiatives that bring together clinicians, data scientists, and managers can bridge knowledge gaps and improve organizational readiness.

### 6.5 Promoting patient-centred digital transformation

AI-based predictive analytics can only succeed in the long term when patients trust and engage actively. Acceptance and legitimacy can be improved with the help of transparent communication strategies and public awareness programs, as well as patient-centric models of consent. To continue the process of trust and to facilitate fair adoption of AI, it is vital to make sure patients have the control over the use of their health data.

## 7 Limitations and future research

Despite its contributions, this study has certain limitations that offer avenues for future research. First, the analysis is a subjective process based on expert judgment inherent in the MCDM methods that can lead to contextual or perceptual bias regardless of the various experts used and consensus building processes. Second, this paper focuses specifically predictive risk analytics in healthcare, which can limit the generalizability of the results to other AI applications, including diagnostics or therapeutic decision support.

Future research could reinforce and extend these findings with the help of large-scale empirical data and real-world implementation outcomes to validate the identified causal relationships. Methodologically, more sophisticated methods as fuzzy DEMATEL, DEMATEL-ANP, or dynamic network-based models of MCDM may be used that would be able to capture uncertainty and changing interdependencies. Comparative cross-country or cross-health-system studies would also provide valuable insights into how regulatory maturity, digital infrastructure, and institutional contexts shape AI adoption readiness.

Together, these future research directions can further refine evidence-based strategies for achieving scalable, ethical, and resilient AI integration across healthcare systems.

## Data Availability

The raw data supporting the conclusions of this article will be made available by the authors, without undue reservation.
